# Nanotopography-based lymphatic delivery for improved anti-tumor responses to checkpoint blockade immunotherapy

**DOI:** 10.7150/thno.35280

**Published:** 2019-10-22

**Authors:** Sunkuk Kwon, Fred Christian Velasquez, John C. Rasmussen, Matthew R. Greives, Kelly D. Turner, John R. Morrow, Wen-Jen Hwu, Russell F. Ross, Songlin Zhang, Eva M. Sevick-Muraca

**Affiliations:** 1 Center for Molecular Imaging, University of Texas Health Science Center, 3Department of Pathology and Laboratory Medicine, McGovern Medical School, Houston Texas 77030; 2 Department of Pediatric Surgery, University of Texas Health Science Center, 3Department of Pathology and Laboratory Medicine, McGovern Medical School, Houston Texas 77030; 3 Department of Melanoma Medical Oncology, M.D. Anderson Cancer Center, Houston, Texas 77030; 4 Kimberly-Clark, Corporation, currently at Sorrento Therapeutics, San Diego, CA; 5 Department of Pathology and Laboratory Medicine, McGovern Medical School, Houston Texas 77030

**Keywords:** Immunotherapy, Nanotopography, Lymphatic delivery, Fluorescence imaging

## Abstract

**Rationale**: Cytotoxic T-lymphocyte-associated antigen 4 (CTLA*-*4) is a co-inhibitory checkpoint receptor that is expressed by naïve T-cells in lymph nodes (LNs) to inhibit activation against “self” antigens (Ags). In cancer, anti-CTLA-4 blocks inhibitory action, enabling robust activation of T-cells against tumor Ags presented in tumor draining LNs (TDLNs)*.* However, anti-CTLA-4 is administered intravenously with limited exposure within TDLNs and immune related adverse events (irAEs) are associated with over-stimulation of the immune system.

**Methods**: Herein, we first deliver anti-CTLA-4 in an orthotopic mammary carcinoma murine model using a nanotopographical microneedle-array device to compare its anti-tumor response to that from systemic administration. Additionally, to demonstrate the feasibility of lymphatic delivery in humans using the device, we use near-infrared fluorescence imaging to image delivery of ICG to LNs.

**Results**: Our data show that lymphatic infusion results in more effective tumor growth inhibition, arrest of metastases, increased tumor infiltrating lymphocytes and complete responses when compared to conventional systemic administration. In clinical studies, we demonstrate for the first time that nanotopographic infusion can deliver ICG through the lymphatics directly to the axilla and inguinal LNs of healthy human volunteers.

**Conclusion**: Taken together, these results suggest that regional delivery using a nanotopography-based microneedle array could revolutionize checkpoint blockade immunotherapy by reducing systemic drug exposure and maximizing drug delivery to TDLNs where tumor Ags present. Future work is needed to determine whether lymphatic delivery of anti-CTLA-4 can alleviate irAEs that occur with systemic dosing.

## Introduction

**Figure 1:**
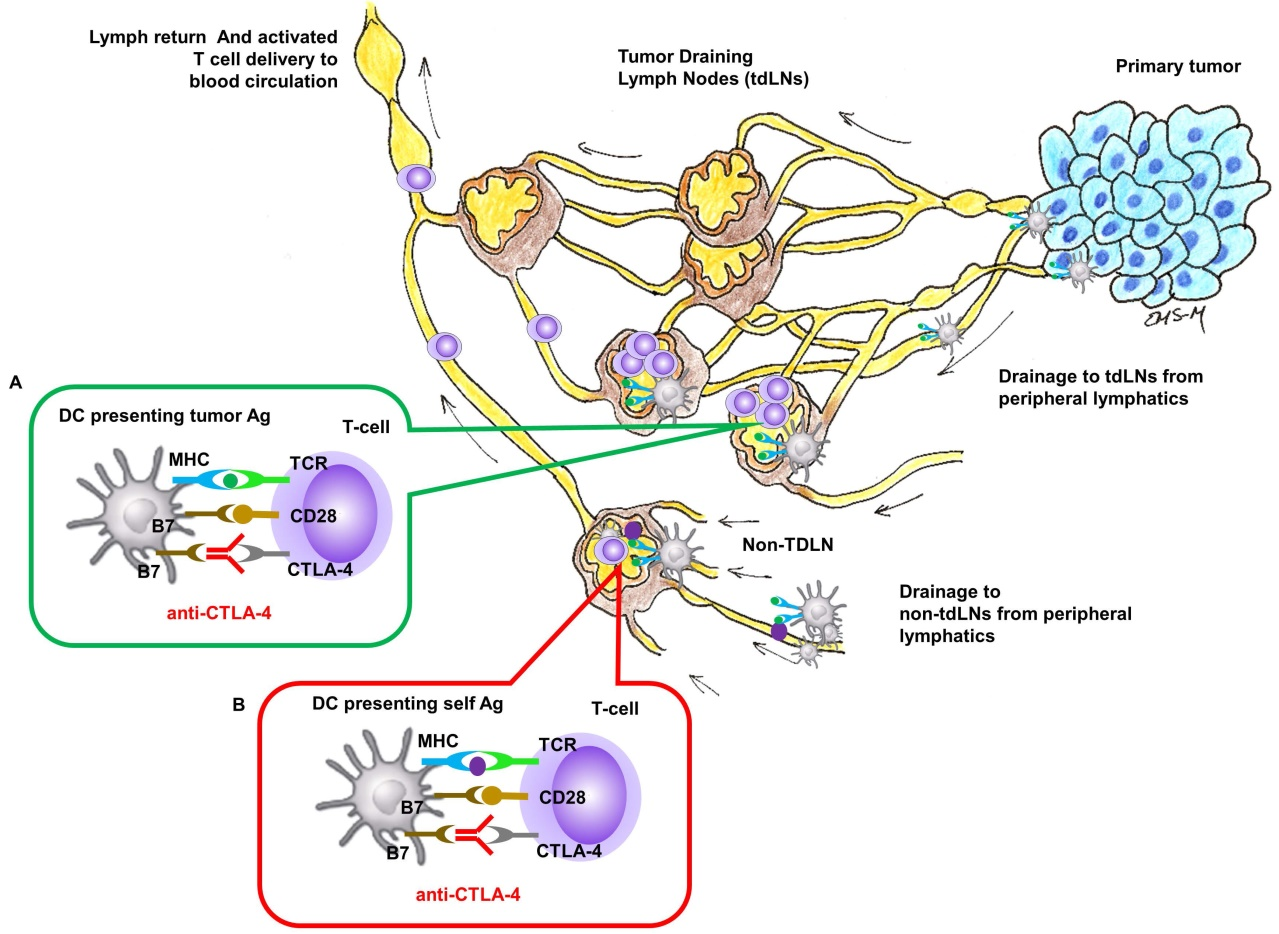
** Site of CTLA-4 as a drug targets for locally advanced metastatic cancer.** Insert **(A)** In tumor-draining LNs, T-cell activation to APC cells presenting tumor Ag can be inhibited by CTLA-4 and blocking inhibition by anti-CTLA-4 could result in T-cell activation against tumor Ag. Insert **(B)** In regional or distant LNs where tumor Ag may not be presented, anti-CTLA-4 blocking of inhibition against T-cell activation to APC cells presenting self Ag response can create irAEs.

Nivolumab or pembrolizumab (targeting programmed death 1 [PD-1] checkpoint monoclonal antibody) and ipilimumab (targeting cytotoxic T-lymphocyte-associated antigen 4 [CTLA-4] checkpoint monoclonal antibody) have become standard immune therapy for patients with advanced cancers. CTLA-4 is expressed on T-cell surfaces and interacts with tumor antigen (Ag) presenting cells (APCs) to suppress the activation of naïve T-cells in lymph nodes (LNs) (Figure 1). While anti-CTLA-4 has also been shown to modify the immune status of the tumor microenvironment 1, the intrinsic site of therapeutic action for anti-CTLA-4 in locally advanced metastatic cancers remains in the tumor draining LNs (TDLNs) where naïve T-cells are activated against tumor Ags.

Both anti-PD-1 and anti-CTLA-4 immunotherapies are administered intravenously (i.v.) and have been shown to induce anti-tumor responses in patients with cancers, including melanoma, non-small cell lung cancer, and renal cell carcinomas. Because anti-CTLA-4 monotherapy is associated with lower response rates and higher rates of severe Grade 3-4 toxicities than anti-PD-1 monotherapy 2, 3, anti-PD-1 monotherapy has become the preferred immunotherapy therapy in patients with advanced melanoma 4. Yet even for immunogenic melanoma, only 50% of patients are responsive to anti-PD-1 monotherapy. For these patients, combination of anti-CTLA-4 and anti-PD-1 therapies have been shown to have complementary activity of up to 50-60% response rates in advanced Stage III or IV melanoma, but, disappointingly, they act synergistically to amplify immune-related adverse events (irAEs) and severe toxicity in up to 60% of all patients 3, 5. Using analysis of outcome data from the CheckMate-067 trial, Oh, *et al*. 6 argue that the elevated costs associated with irAEs due to anti-CTLA-4 in combination with anti-PD-1 render it cost-ineffective despite the benefits of improved disease free survival in responsive cancers. Maximizing exposure of anti-CTLA-4 in TDLNs where cytotoxic T cells are activated against tumor Ags may improve anti-tumor responses and minimize dose dependent irAEs.

LNs are part of the open, unidirectional lymphatic vasculature. The entry point for capillary filtrate, macromolecules, and immune cells is at the “initial” lymphatics that (i) lie immediately below the epidermis, (ii) surround the periphery of all organs, and (iii) can be formed at the tumor periphery through the process of tumor lymphangiogenesis (Figure 1). These “initial lymphatics” are immature capillaries without a basement membrane and with loose endothelial cell tight junctions that open and close to uniquely allow entry of waste materials and immune cells. From the initial lymphatics, lymph drains through mature, conducting lymphatic vessels that consist of a series of vessel segments bounded by valves and lined with smooth muscle cells that contract to actively propel lymph (often against gravity) to regional LN basins. Following transport through chains of downstream LNs via afferent and efferent lymphatic vessels, lymph is deposited into the blood vasculature via the subclavian vein 7. Monoclonal antibody access to the regional lymphatics following i.v. administration can occur through the high endothelial venules (HEVs) of LNs that are exclusive entryways for naïve T and B cell entry. In addition, antibodies that have extravasated from the blood vasculature may also be taken up by initial lymphatics for delivery to regional LNs. However, because lymph drains into the blood vasculature, comparatively large i.v. doses may be needed to reach drug targets associated with tumor Ags presentation within tumor draining LNs (Figure 1 insert A). Systemic, i.v. administration of anti-CTLA-4 can also result in activated naïve-T cells in non-tumor draining LNs where non-tumor, self Ags rather than tumor Ags are presented (Figure 1, insert B), and presumably lead to irAEs. In this work, we hypothesize that lymphatic delivery of anti-CTLA-4 will have greater anti-tumor responses than systemic delivery in a preclinical model of metastatic cancer. Since preclinical models poorly predict irAEs to checkpoint blockade immunotherapy 8, we test for similar or improved tumor responses with lymphatic delivery.

Administration of biologics directly into the lymphatics is challenging, with intradermal (i.d.) or Mantoux administration offering the most accessible entry point into the initial lymphatics below the epidermis. Unfortunately, the small sub-epidermal volume can accommodate ~100 µL and ~ 50 µL of injected fluid in humans and in rodent models, respectively and limits the therapeutic dose of lymphatically delivered biologics in both preclinical and clinical investigations. Deeper, subcutaneous (s.c.) or intramuscular (i.m.) administration below the sub-epidermal space is inefficiently taken up by initial lymphatics and has reduced bioavailability due to off-target drug uptake and cellular processing 9.

**Figure 2:**
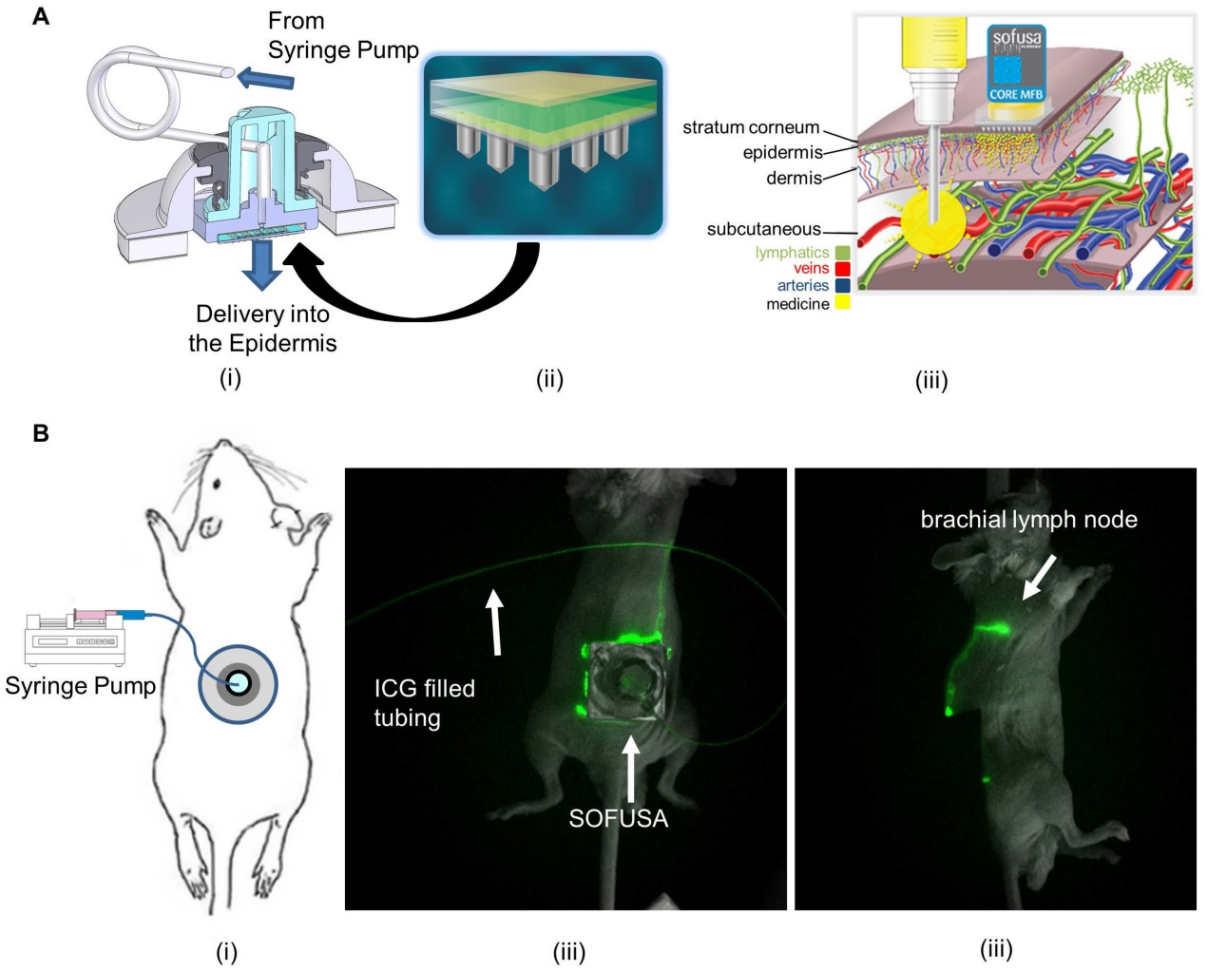
** A.** Schematic of the (i) SOFUSA™ Nanotopographical Device for infusing anti-CTLA-4 into the sub-epidermal space consists of a (ii) microfluidic fluid block with a microfluidic distributor (green) and silicon microneedle array (gray). Each microneedle is 350 μm long, 110 μm wide with a 30 μm through hole located off center which the drug flows out. (iii) SOFUSA^TM^ infuses drug into the sub-epidermal space where initial lymphatic provide uptake, whereas deeper subcutaneous injections deposit drug below the initial lymphatics reducing uptake. **B.** (i) Placement of SOFUSA^TM^ on dorsal back of mice. NIRF (ii) dorsal and (iii) lateral images of SOFUSA^TM^ delivery of ICG to brachial LN. (see Additional file 1: Video 1 showing lymphatic pumping of ICG delivered via SOFUSA^TM^ to the brachial LN). The SOFUSA^TM^ device is covered with black cloth to prevent oversaturation of NIRF imaging system.

In this contribution, we show that the anti-tumor responses of anti-CTLA-4 are significantly improved in a preclinical model when delivered with SOFUSA^TM^, a nanotopography-based lymphatic delivery system described previously by us and others 10, 11. The SOFUSA^TM^ lymphatic infusion device consists of a single-use, 66 mm^2^ array of 100 microneedles of 110 µm diameter, 350 µm long, and with a 30 µm hole located off-center (Figure S1). A polyether ketone nanotopographical film heat-formed over each microneedle provides the nanotopographical features. These features have been shown to reversibly remodel tight junction proteins initiated via integrin binding to the nanotopography structures 10 potentially augmenting uptake into the initial lymphatics. In both animals and humans, the device is first affixed via adhesive onto the tissue site of infusion, and then a calibrated applicator pushes the microneedle array into the sub-epidermal space where the initial lymphatics are located. A calibrated syringe pump then delivers drug into the microfluidic chamber, through the microneedle array, and into the sub-epidermal space in volumes not otherwise achievable through i.d injections (Figure 2A). We hypothesize that SOFUSA^TM^ can deliver substances into draining LNs at least as effectively as i.d. administrations, but with greater volumes than possible by i.d. injection.

In this work, we find that SOFUSA^TM^ delivery of anti-CTLA-4 in an orthotopic 4T1-luc mammary carcinoma murine model results in a greater number of complete responses, significantly reduced tumor growth, and a striking inhibition of distant metastases than when administered systemically. And in order to demonstrate the feasibility of SOFUSA^TM^ nanotopography delivery through lymphatic vessels directly into draining LNs in humans, we use near-infrared fluorescence (NIRF) imaging to show direct delivery of an inert fluorescent agent into axillary and inguinal LN basins. Taking preclinical and clinical results together, this report suggests that lymphatic delivery of checkpoint inhibitors involved in the naïve T cell priming phase in the tumor draining LNs could potentially improve anti-tumor responses and alleviate disseminated irAEs in patients with cancers over that currently treated by conventional i.v. infusion.

## Results and Discussion

### Lymphatic delivery of anti-CTLA-4 improves anti-tumor response in an orthotopic mammary carcinoma murine model

In non-tumor bearing animals, NIRF imaging showed that SOFUSA^TM^ can effectively infuse 100 µL/h of ICG into the epidermal spaces wherein the initial lymphatics take up the agent enabling visualization of the propulsion of ICG-laden lymph into the brachial LNs (Figure 2B, Supplemental Video 1) that also receive lymph drainage from the mammary chain. Using these diagnostic studies to understand how SOFUSA^TM^ infusion results in lymphatic delivery, we then tested the system to deliver immunotherapy in tumor bearing mice. In BALB/C mice with orthotopic implants of 4T1-luc mouse mammary in the right caudal mammary fat pad, SOFUSA^TM^ was used to infuse 10 mg/kg anti-CTLA-4 (clone 9H10, BioXCell) in 0.05 mL PBS on the right lateral side with infusion rates of 100 µL/h on days 11, 15, 19, and 23 post implant (p.i). Tumor growth rates and, in a subset of animals, bioluminescence imaging of tumor burden were compared to additional groups of tumor bearing animals receiving either 10 mg/kg anti-CTLA-4 or isotype control antibody (Polyclonal Syrian Hamster IgG, BioXCell) through intraperitoneal (i.p.) injection on days 11, 15, 19, and 23 p.i.

**Figure 3:**
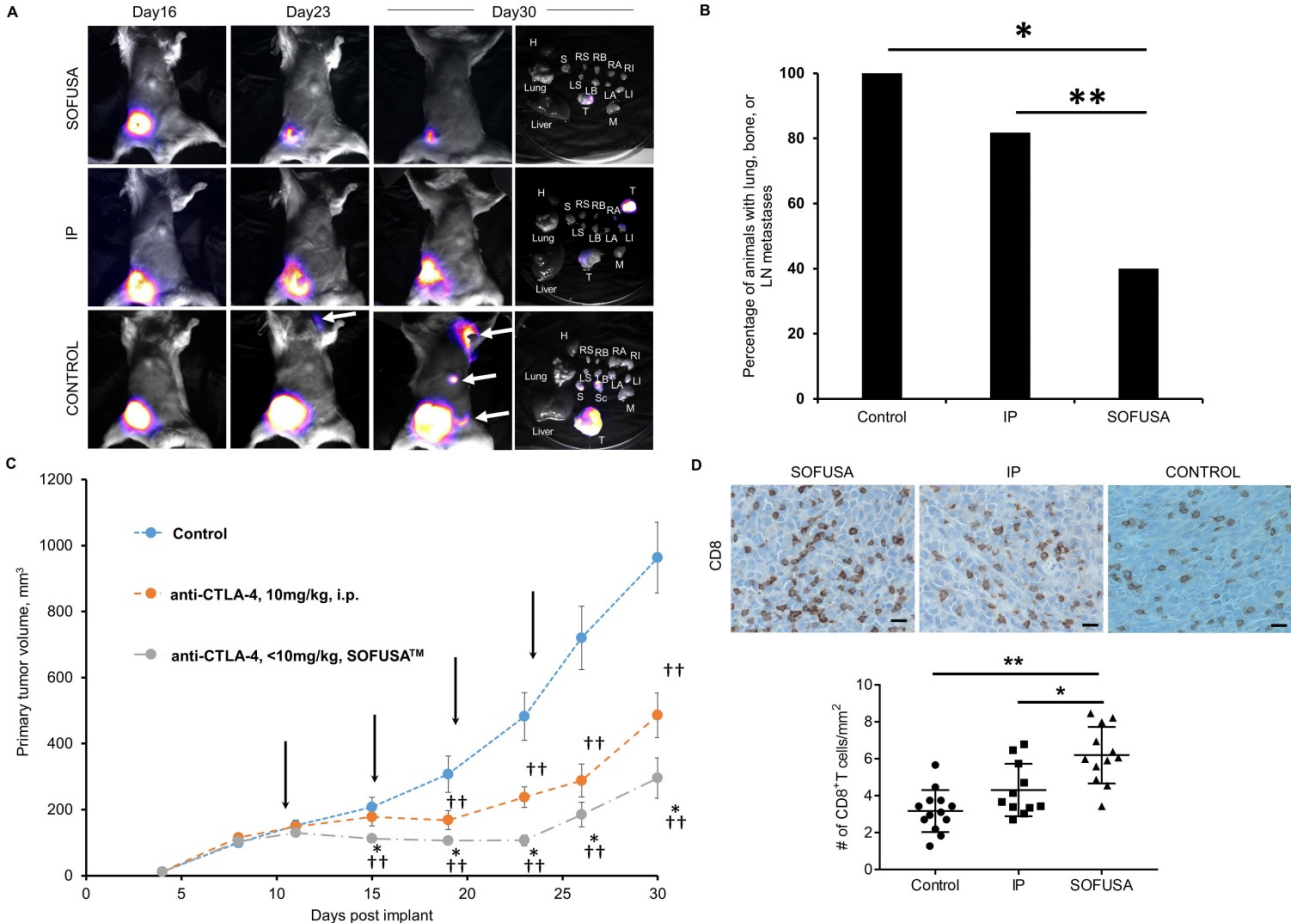
** A.** Example bioluminescence images of orthotopic 4T1-Luc in Balb/c mice 16, 23, and 30 days after tumor inoculation and tissues after euthanasia. CTLA-4 was treated i.p or via SOFUSA^TM^ at 11, 15, 19, and 23 days post implantation. Examples of animals with complete and partial response to SOFUSA^TM^ infusion of anti-CTLA-4 are illustrated along with animals with systemic i.p. administration of anti-CTLA-4 and isotype control antibody. H, heart. RS/LS, right/left submandibular LN. RB/LB, right/left brachial LN. RA/LA, right/left axillary LN. RI/LI, right/left inguinal LN. T, tumor. M, muscle. S, stratum. Sc, scapula. White arrows indicate distant metastases and images are scaled differently to highlight presence of metastases. Supplemental images of additional animals are provided in Figure S2. **B.** Percentage of animals with lung, LN, or bone metastases in animals receiving isotype control systemically (N=13) and receiving anti-CTLA-4 systemically (N=11) or via SOFUSA^TM^ infusion (N=15). There is statistically reduced tumor burden in SOFUSA^TM^ dosed animals compared to animals systemically dosed with anti-CTLA-4 or isotype control antibody (z-test). * p<10^-5^. ** p<0.01. C. Average growth rates +/- SE of orthotopic 4T1-Luc in Balb/c mice treated with control antibody (blue; n=17) or with anti-CTLA-4 (orange; n=11)) administered i.p., or with SOFUSA^TM^ delivered anti-CTLA-4 (grey; n=18). Arrows indicate treatment days. (*) denotes statistical differences (p≤0.05) in tumor volumes in animals receiving anti-CTLA-4 via SOFUSA^TM^ infusion and i.p. injection. (†, ††) denote statistical differences between tumor volumes of animals treated with isotype control or with anti-CTLA-4 administered via lymphatic infusion or systemic injection. The mean tumor volumes ± SE (bars) are shown at the times that tumor measurements were made. D. Representative primary tumor tissues from each group were immunostained for CD8a. Scale, 10 µm. In the animals evaluated with bioluminescence, the number of CD8 positive T cells per mm^2^ was measured in the mice treated with control antibody (N=13), systemic administration of anti-CTLA-4 (N=11), and SOFUSA^TM^ administration of anti-CTLA-4 (N=12). ** p<0.0001, *, p=0.0062. Tissues from three animals dosed with SOFUSA^TM^ were not available at endpoint due to complete responses or inadequate residual tissue.

Bioluminescence imaging in a subset of animals showed anti-CTLA-4 arrested tumor growth and LN, bone, and lung metastases at day 23 and 30 p.i., and from ex-vivo imaging at the 30 day study endpoint, the amount of distant metastases (Figure 3A). Of the animals dosed with SOFUSA^TM^ infusion, 22% (4/18) possessed a complete response as determined by undetectable primary tumor volume by caliper measurement. No animals receiving i.p. administration of anti-CTLA-4 (0/11) or isotype control (0/17) exhibited these complete responses, suggesting that lymphatic delivery to TDLNs potentiated early and robust anti-tumor activity. In addition, there were statistically significant less animals with distant metastases in SOFUSA^TM^ infused animals compared to systemically dosed and isotype control groups (p<0.01), but no difference in between the systemically dosed and isotype control groups. In the subset of animals imaged by bioluminescence for detection of distant metastases, 100% (13/13) of animals in the isotype control group, 82% (9/11) in the systemically dosed group, and 40% (6/15) of those dosed via SOFUSA^TM^ infusion exhibited distant metastases to the bone or LNs, with lung metastases being the most prevalent. These results suggest that despite the regional delivery of drug, there is an effective abscopal anti-tumor response from regional SOFUSA^TM^ dosing in comparison to systemic dosing. This result is consistent with (i) the greater drug exposure to naive T cells in TDLNs with lymphatic delivery and (ii) a more effective activation of T-cells against tumor Ags which once educated, leave TDLNs to mount a robust systemic anti-tumor response. Of the subset of animals with lung metastases, there were statistically (p<0.01) fewer number of lung lesions in animals dosed with anti-CTLA-4 compared to isotype control, but no difference arose from animals receiving anti-CTLA-4 via differing routes of administration, i.e. lymphatic versus systemic administration. While lymphatic delivery increased the number of animals with complete responses, in animals with incomplete or no response, lymphatic delivery of anti-CTLA-4 was no less effective than when administered systemically.

As shown in the group-averaged data of tumor growth in Figure 3C, tumor bearing animals receiving SOFUSA^TM^ infusion of anti-CTLA-4 exhibited significantly reduced tumor growth at day 15 p.i. and onwards when compared to animals dosed with isotype control antibody. Tumor bearing animals receiving i.p. injection of anti-CTLA-4 exhibited statistically significant reduced tumor growth on day 19 p.i. and onwards when compared to animals administered with isotype control antibody. Beginning at day 15 p.i., the tumor volumes of animals having received the first round of anti-CTLA-4 via SOFUSA^TM^ infusion were significantly smaller than those who received drug systemically. This data suggests an earlier anti-tumor response in animals dosed regionally with SOFUSA^TM^ infusion into the lymphatics as compared to those receiving drug systemically. IHC staining of the subset of SOFUSA^TM^-dosed animals that had residual primary tumor at study endpoint, showed statistically greater number of tumor infiltrating lymphocytes (TILs) in the primary tumors compared to controls (p<0.0001) or to animals dosed systemically (p=0.006; Figure 3D). Given that TILs are associated with enhanced clinical responses to ipilimumab 12, these results are consistent with improvement in response due to increased exposure to drug target in the TDLNs. Interestingly, there was no statistical difference between TILs in animals receiving systemic administration of control or CTLA-4 antibody. It is important to note that in animals receiving anti-CTLA-4 systemically and by SOFUSA^TM^, rebound of tumor of tumor growth occurs in a portion of the animals, consistent with reports of monotherapy in other studies.

If lymphatic delivery of anti-CTLA-4 to TDLN reduces the dose-dependent irAEs observed in humans 13, then combinational therapies that are otherwise too toxic for use in many patients 5, 14 could potentially increase the anti-tumor response as seen in this preclinical study. It is noteworthy that the 4T1 mammary carcinoma model used herein is considered non-immunogenic and non-responsive to anti-CTLA-4 or anti-PD-1 monotherapy, but responsive to combination therapy 15, 16. While the luciferase protein may confer immunogenicity of the tumor line and explain the partial responsiveness to anti-CTLA-4 monotherapy seen herein, further studies remain to determine whether increase responsiveness as seen with systemically delivered combinational therapies can be achieved using lymphatically delivered combinational therapies. As with CTLA-4, PD-1 is expressed by T-cells in TDLN, and associates with its ligand, PD-L1 expressed on the lymphatic endothelium 17 and on tumor cells to limit T cell effector function 18 in the tumor microevironment. Lymphatic delivery of anti-PD-1 could selectively remove mechanisms for inducing tolerance to tumor Ags within the TDLN, but, since lymph ultimately empties in the blood circulation, lymphatic delivery could also restrict tolerance acquisition at peripheral and tumor sites. Finally, while this study utilized hamster rather than a potentially more relevant mouse derived IgG against mouse CTLA-4, further studies are needed to determine whether antibody isotype impacts anti-tumor response.

While our previous studies used radioactivity balances in rats to show that SOFUSA^TM^ infusion provided delivery as accurately as conventional hypodermic syringes 11, the small size (20-25 g) of mice employed in this study unfortunately resulted in incomplete seeding of the entire microneedle array into the epidermis on the dorsal lateral sides, resulting in leakage of drug and incomplete dosing. The total volume infused was estimated by using a syringe to collect drug on the skin at the end of the infusion, and subtracting the volume collected from the total volume delivered by the calibrated syringe pump. Using this approach, we estimate between 30% and 100% of the systemic dose (0.06 - 0.2 mg of drug) with an average of 74 ± 24% of the total intended dose was infused, yet found no correlation between the estimated dose infused via SOFUSA^TM^ and the measured anti-tumor responses. This limitation in SOFUSA^TM^ dosing may be overcome by designing and using a smaller microneedle array specific for preclinical studies. Despite this preclinical limitation of receiving less drug, animals lymphatically dosed with anti-CTLA-4 experienced greater anti-tumor responses than those animals systemically dosed at higher levels, which is consistent with greater exposure to targets with lymphatic delivery. Whether lymphatic delivery of even smaller amounts of drug can achieve equivalent anti-tumor responses as systemically delivered drug remains to be tested in a lymphatic dosing study with more accurate delivery. Due to enhanced exposure to drug targets, lymphatic delivery may allow a dramatic reduction of dose and may potentially reduce the current i.v., dose-dependent irAEs that limit clinical use of ipilimumab 19 and other emerging immune checkpoint inhibitors. Nonetheless, the safety of lymphatic delivery of ipilimumab will need to be clinically evaluated, since increased drug exposure to targets and non-targets may have unintended consequences.

There were several limitations of our studies that could be improved upon in future studies. First, the use of a single study endpoint did not enable temporal or more detailed mechanistic evaluation of immune responses between systemic and lymphatic route of administrations. Future work will need to evaluate survival as well as early time immune responses in TDLNs, contralateral LNs, blood, and in the tumor microenvironment. This work will also need to be expanded into non-immunogenic, immunogenic, and transgenic models of orthotopically implanted and spontaneous tumors, and potentially implemented with combinational therapies, including therapeutic vaccination. Finally, testing in models with transient depletion of regulatory T cells (Tregs) 20 will provide evidence whether lymphatic delivery can reduce irAEs in addition to enhancing anti-tumor immunity.

### Near-infrared fluorescence imaging of ICG shows lymphatically directed delivery to draining LNs

**Figure 4:**
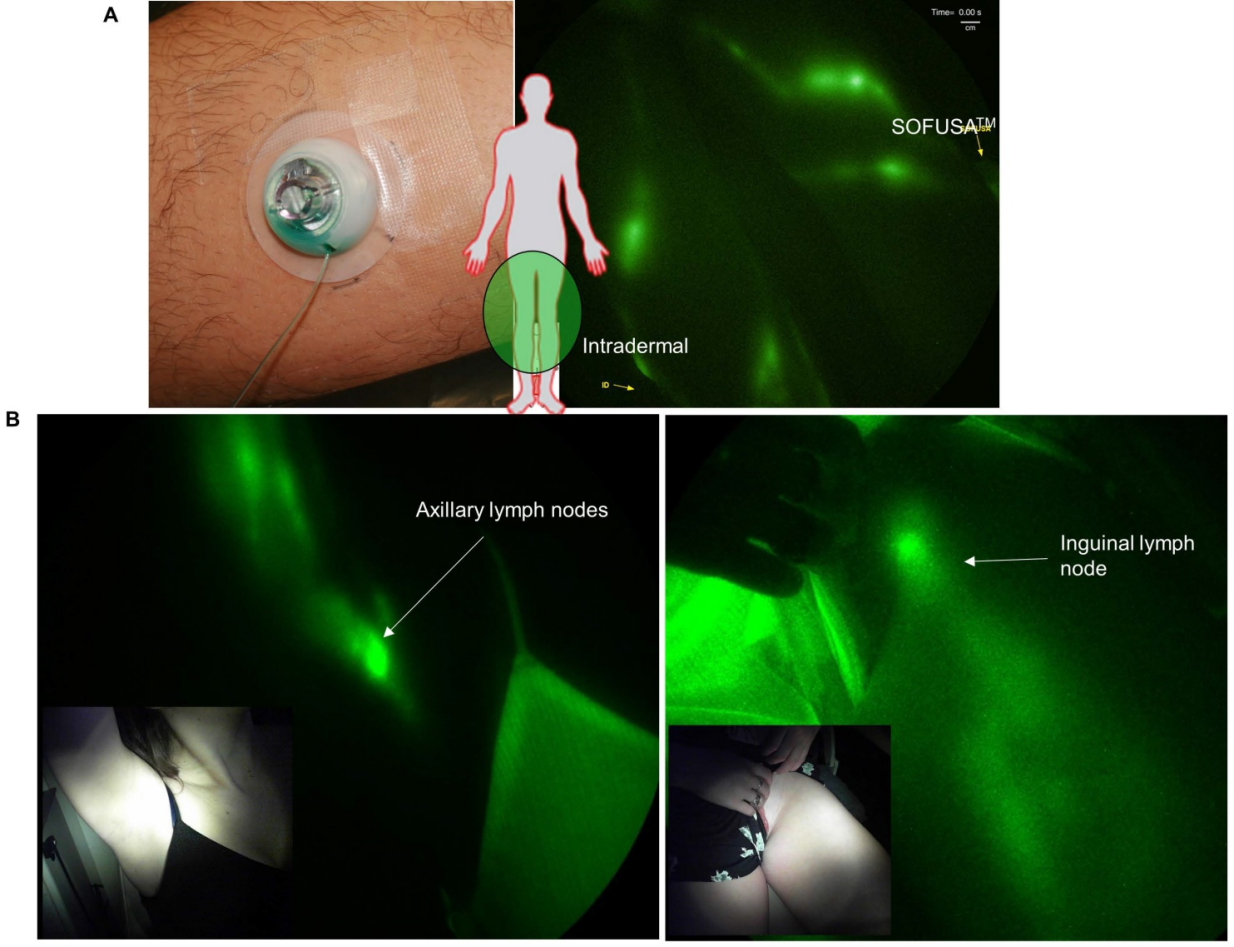
** A.** Left: Applied SOFUSA^TM^ device infusing ICG and Right: NIRF imaging of lymphatic vessels propelling ICG-laden lymph (Supplemental Video 2) during infusion (right) and i.d. injection (left) of ICG in the medial ankle and lateral calf with inserts showing i.d. injection and SOFUSA^TM^ infusion placement. **B.** Near-infrared fluorescence images of SOFUSA^TM^ delivery of ICG into the axilla and inguinal LNs of healthy volunteers.

**Figure 5:**
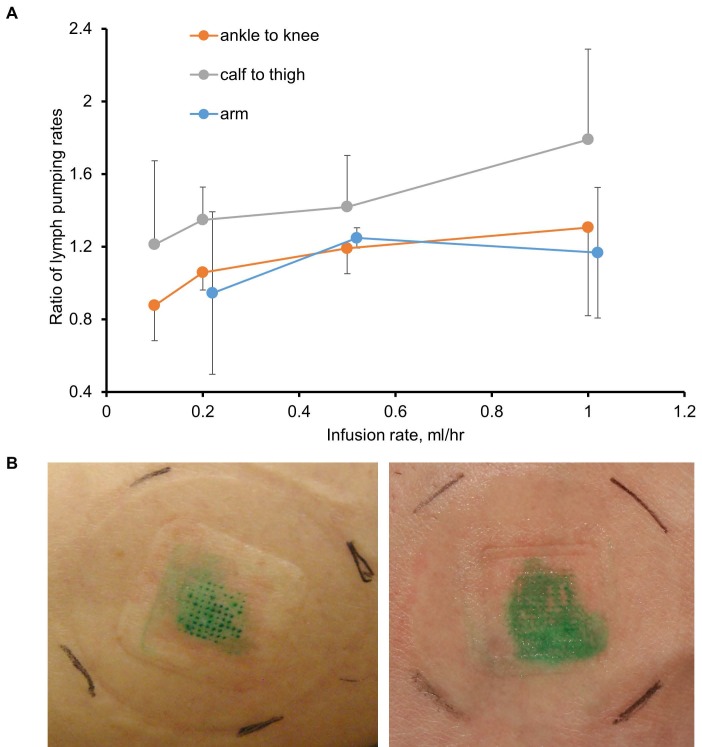
** A.** The average ± SE ratio of lymphatic contractile pumping in lymphatic vessels draining SOFUSA^TM^ infusion sites to that in vessels draining from contralateral i.d. injections sites as a function of infusion flowrate for administration sites in the arm, ankle, and calf. **B.** Photographs of tissue sights after removal of SOFUSA^TM^ device that infused ICG at rates of less than 1 mL/h (left) and at 1 mL/h (right) with the latter showing “pooling” of ICG in the epidermis.

Before SOFUSA^TM^ technology can be considered for infusion of checkpoint blockade immunotherapies in cancer patients, its feasibility for lymphatic delivery needs to be assessed in human subjects. In a pilot study of 12 human volunteers, we showed SOFUSA^TM^ is capable of delivering drug to LNs as shown through NIRF lymphatic imaging of ICG. In the first 8 subjects, device placement and microneedle penetration were optimized for infusions on the dorsal aspect of the wrist, lateral aspect of ankles, and medial aspect of calf and could be achieved with less success on the dorsal foot. In subjects of low BMI, the placement on the foot and wrist were often complicated with incomplete penetration of microneedles into the dermis as visualized by ICG leakage and impaired uptake. Figure 4A (and Supplemental Video 2) shows the lymphatic vessels imaged following SOFUSA^TM^ infusions and contralateral i.d. injections in the medial aspect of the calf and the expected symmetry of functional lymphatic vessels that pump ICG-laden lymph to the regional axillary and inguinal LNs (Figure 4B). With subjects sitting upright, we found lymphatic pumping rates expectedly ranged between 0.4 - 3.3 min^-1^, consistent with past work 21. The lymphatic pumping rates of ICG-laden lymph resulting from SOFUSA^TM^ infusion were consistently faster when ICG was infused at rates > 0.2 mL/h with SOFUSA^TM^ than when delivered via i.d. injection at the contralateral site. In addition, as shown in Figure 5A, the ratio of lymphatic pumping rates resulting from SOFUSA^TM^ infusion to that resulting from i.d. administration also tended to increase with SOFUSA^TM^ infusion rates, but, due to the small sample size, no further analysis or statistical significance could be determined. Whether the nanotopographic features on the microneedle array are responsible for the enhanced filling and increased active transport remain to be investigated. However, previous data in animals showed the nanostructured microneedle array devices cumulatively delivered significantly more etanercept than microneedle array devices without nanostructured coating by measuring the appearance of drug in the serum of rats and rabbits 10. At SOFUSA^TM^ infusion rates of 1 mL/hr, ICG “pooling” at the infusion site was visualized after removal of the device, indicating that the lymphatic uptake in the initial lymphatics in the sub-epidermal space was slower than the infusion rate. Figure 5B shows the transient demarcations of ICG left by the microneedle array immediately after removal when there was no “flooding” of the sub-epidermal space. After 24 hours, these demarcations disappear. Subjective assessment of pain owing to application of SOFUSA^TM^, infusion, and removal of SOFUSA^TM^ device was performed for each device application using a visual analog scale (VAS) questionnaire with a range of 0 - 100 with the value of 0 associated with no discomfort sensation and with the value of 100 associated with extreme pain. The average ± SD VAS pain score for application, infusion, and removal of the SOFUSA^TM^ was 8 ± 9, 5 ± 8, and 1 ± 4, respectively, indicating that the device caused between no pain to mild pain 22. There were no adverse events associated with the ICG or the SOFUSA^TM^ device during the time of the study or at study follow-up, which occurred 24 hours after the study.

### Prospects for lymphatic delivery of immunotherapy in cancer patients

SOFUSA^TM^ infuses drug within the sub-epidermis space and therefore accesses capillaries of both the hemovascular and lymphovascular systems. Many factors including size, composition, dose, surface charge, and molecular weight affect uptake into lymphatic and/or blood capillaries. For example, large particles, immune cells, and macromolecules are primarily taken up by lymphatic capillaries, while small particles and molecules less than 20 kDa can be absorbed by blood capillary networks 23. More recently, Levick and Michel 24 recently showed that the glycocalyx on the luminal sides of blood vessels and capillaries is responsible for a force opposing capillary pressure and inhibits re-absorption of fluid into the venous vasculature. Thus while the blood capillaries are intact and comparatively impermeable in the sub-epidermal space, the “initial lymphatics” represent immature capillaries without a basement membrane. They have “loose” lymphatic endothelial cell tight junctions that open and close via fibrils to uniquely allow entry of macromolecules, waste products, and immune cells. In fact, it has been estimated that as much as 12 liters of capillary filtrate (carrying small solutes and macromolecules) is collected from peripheral tissues by the initial lymphatics and returned to the blood vasculature 25. Because lymph drains to the blood vasculature, pharmacokinetic profiling in serum as performed in past studies of SOFUSA^TM^ delivery provides 10, 11 a measure of effectiveness of delivery through the lymphatics to the blood vasculature.

While we have previously labeled monoclonal antibodies with near-infrared fluorophores for visualizing drug trafficking and distribution 26, non-specific labeling can impact immunoreactivity. Therefore, we did not conduct labeling in our preclinical studies to demonstrate therapeutic effect. Instead, we compared SOFUSA^TM^ infusion of ICG with i.d. injections that are known to routinely drain to lymphatic basins 27, 28. Our studies show that, for the first time, SOFUSA^TM^ infusion mimics i.d. injections in humans (albeit at larger volumes) and suggests that lymphatic delivery of immunotherapies in cancer patients should be possible. Our preclinical results suggest that by delivering drug *through* the lymphatics, drug exposure to targets that reside within lymphatics are maximized for more effective anti-tumor responses.

Maximizing the exposure of immune checkpoint therapy to naïve T cells in the presence of tumor Ags while preventing T cell activation against non-tumor self-Ags is a strategy that could improve treatment efficacy as well as safety of cancer immunotherapies. Similar strategies have focused on (i) i.v. administration of targeted nanoparticles that deliver immunotherapies/immunmodulators to cancers or to subsets of endogenous immune cells within the blood circulation 29-31, (ii) nanoparticle-loaded, degradable microneedle patch-assisted or scaffold-based sustained delivery of immunotherapies 32, 33, (iii) i.d. injection of nanoparticle agents that induce dendritic cell maturation and vaccination within tumor draining LNs 34, or (vi) intratumoral or peritumoral injections of immunotherapies that eventually drain to tumor draining LNs 35. In the latter case, currently approved immunotherapies may require infusion volumes that may be too large for peritumoral or intratumoral injections that in themselves may not always be clinically safe and feasible. Nanoparticle formulation of immunotherapeutics add complexity that could limit clinical translation, while phagocytosis of nanoparticle formulations of extracellular agonists and antagonists for T-cell priming in the LNs may restrict or limit drug exposure to extracellular targets. In contrast, lymphatic delivery devices such as the nanotopographic SOFUSA^TM^ microneedle-array device used herein could employ conventional formulations of approved drugs albeit at a potentially lower dose because of the maximized drug exposure to immune cells that have high chance to experience tumor Ag presentation in TDLNs.

It is noteworthy that the impact of lymphatic delivery on tumor responses seen in this preclinical study may be attenuated in the small, quadrupedal preclinical tumor models as compared to bipedal non-human primates or patients. Systemic administration of monoclonal antibodies in rodent studies are commonly performed with i.p. injection for effective uptake by the plentiful lymphatics in the peritoneal cavity that promptly empties into the venous system. As a result, i.p. administration largely approximates the same pharmacokinetic and pharmacodynamic profiles seen with i.v. injection. While i.p. administration may escape the exposure to tumor draining LNs seen in this lymphatic delivery study, the i.p. route of administration nonetheless uses the truncal lymphatics to deliver drug to the blood circulation. As a result of the exposure in the lymphatic compartment, the anti-tumor responses from i.p. administration in rodents may be expected to overpredict those from i.v. administration in humans. In addition to the attenuated response in rodent models, adverse immune responses to immunotherapies are generally non-existent in rodents, further requiring clinical investigation to understand whether lymphatic delivery can ameliorate irAEs that limit combinational and emerging agonist immunotherapies. Translational studies of lymphatic delivery of immune modifying drugs will need to address (i) the heterogeneity of lymphatic anatomy in humans, (ii) lymphatic re-routing and ectopic LNs that are known to occur with advanced disease, and (iii) the pragmatic difficulties of recruiting advanced cancer patients with intact TDLNs, especially since LN dissection is often standard-of-care treatment during initial diagnosis. Interestingly, the standard-of-care for diagnosing, staging, and treating of many cancers is LN dissection, which itself involves the removal of the very TDLNs that harbor the targets for current and emerging cancer immunotherapies. Indeed, Fransen and colleagues 36 recently demonstrated in preclinical models that TDLNs are essential for efficacy of PD-1/PD-L1 checkpoint therapy. While the development of lymphatically-directed immunotherapy could revolutionize the management of cancer, several technological issues remain to ensure the safety and effectiveness of treatments. The tolerizing properties of lymphatic endothelial cells in efferent lymphatic vessels, the determinants of the active contractile lymphatic propulsion necessary for afferent delivery of drug, and the timeframe for acquisition of anti-drug antibodies in response to drug exposure in LNs, will all need to be elucidated before lymphatic delivery of immune-modifying drugs can be routinely performed and used as an accepted route of administration. In addition, there is no current ability to routinely sample lymph for assessment of drug concentration and the non-circulatory, unidirectional lymphatic vasculature escapes modeling as a compartment for pharmacokinetic and pharmacodynamic predictions. Deciphering the biomarkers of regional immune activation for systemic anti-tumor immunity will be essential to validate and establish the effectiveness of lymphatic delivery of cancer immunotherapies with targets of T-cell priming. Hence before lymphatic delivery can be ethically translated in cancer patients, unique strategies for combining therapy with diagnostics, i.e., theranostics, will need to be developed.

## Methods and Materials

All animal studies were conducted following approval from the University of Texas Health Science Center Animal Use Committee and human studies were conducted following approval from the University of Texas Health Science Center Institutional Review Board. The human studies employed off-label administration of ICG through the SOFUSA^TM^ device under approved FDA combinational IND 136,057.

*Orthotopic 4T1 animal model and immunotherapy treatment* 5 x 10^5^ luciferase-transfected 4T1 (4T1-luc) mouse mammary tumor cells (37, kindly provided by Mien-Chie Hung, Ph.D., The University of Texas MD Anderson Cancer Center, Houston, TX) in 0.1 mL of PBS and Matrigel were injected into the right caudal mammary fat pad of BALB/C mice. At day 11, animals were separated into one of three treatment groups that received (1) 10 mg/kg anti-CTLA-4 (clone 9H10, BioXCell) in 0.05 mL PBS i.p.; (2) 10 mg/kg anti-CTLA-4 in 0.05 mL PBS infused via SOFUSA^TM^ ; and (3) 10 mg/kg isotype control antibody (Polyclonal Syrian Hamster IgG, BioXCell) on days 11, 15, 19, and 23 p.i. All the cohorts had similar tumor volumes at start of dosing on day 11. Animals with tumor volumes that were statistically different from the group at day 11 were not included in the analysis. Study endpoint was 30 days post-implant or the tumor exceeds 20 mm in any dimension, whichever came first.

*SOFUSA^TM^ lymphatic infusion device in preclinical studies* In animals, 50 µL of 4.5 mg/mL solution of anti-CTLA-4, and 50 µL of 0.5 mg/mL ICG were infused over an hour on the right dorso-lateral side of isoflurane anesthetized animals. Lymphatic imaging was performed non-invasive near-infrared fluorescence imaging as previously described 27.

*Assessment of tumor burden* At days 4, 8, 11, 15, 19, 23, 26, and 30 days post-implant (p.i.), short (D1) and long (D2) tumor dimensions were assessed from caliper measured and volumes (V) computed from 0.5 x D1^2^ x D2 38. At days 16, 23, and 30 days p.i. tumor burden was assessed in a subset of animals using bioluminescence with a custom build, bioluminescence device. *In vivo* bioluminescence images were acquired 10 min after i.p. administration of D-luciferin (150 mg/kg in 200 µL of PBS; Goldbio). For *ex vivo* bioluminescence imaging at 30 days p.i., organs were removed immediately after the second D-luciferin administration (approximately 20 min after the first D-luciferin injection), incubated in D-luciferin solution, and imaged. Tissues were subsequently evaluated through gross examination and histology.

*Immunohistochemical staining Tissue* samples were embedded in paraffin and 4 μm sections used in all staining procedures. Following paraffin removal and antigen retrieval using citrate buffer, tissues were incubated with H_2_O_2_, blocked with 5% normal goat serum albumin, and stained with rat anti-mouse CD8 antibody (eBioscience^TM^) and biotin-anti rat secondary antibody (Vector Labs). The Vectastain Elite ABC system for peroxidase and DAB as chromogens were used before tissues were counter-stained with hematoxylin (Vector Labs). CD8 expression was examined at x63 magnification (Zeiss Axio).

*SOFUSA^TM^ lymphatic infusion device in clinical studies* In a pilot study of 12 normal humans 0.25 mg/mL solution of indocyanine green (ICG) was infused lymphatically for a period of 60 min using a calibrated infusion pump (Model 4100, Atlanta BioMedical Corporation), and the nanotopographical device positioned on the dorsal aspect of feet, lateral aspect of ankles, medial aspect of calf, and/or the wrist. The uptake of ICG was monitored using a custom built near-infrared fluorescence imaging system employing a Gen III GaAs intensifier coupled to a sCMOS 39 to visualize delivery to inguinal and axillary LNs, and to quantify lymphatic propulsion and transport of ICG-laden lymph at contralateral sites after i.d. injection. Optimization of device placement and microneedle tissue penetration was performed on the first 8 subjects. In the last four subjects, infusion rates were varied between 0.2 - 1 mL/h and afterwards, lymphatic propulsion was analyzed from acquired images by counting the number of ICG-laden lymphatic “packets” that crossed a chosen anatomical landmark. Lymphatic pumping rates resulting from infusion were compared to those from the contralateral locations where ICG was administered intradermally. Contralateral i.d. injections were made using a conventional insulin syringe and 31 gauge needle to deliver 0.1 mL of 0.25 mg/mL ICG solution and were often made following desensitization with cold spray for those volunteers who were sensitive to needle prick. Cold spray was not used for application of the SOFUSA^TM^ infusion device and pain was assessed via a visual analog scale (VAS) questionnaire for each infusion device applied. Up to five different devices were placed on each volunteer for simultaneous infusion.

*Data analysis and statistics* Tumor growth data is presented as average volumes ± standard error (SE). Statistical analysis was performed with Microsoft Excel and volume data from individual time points were analyzed by unpaired 1-tailed Student's t-test with the significance level set at p≤0.05. Upon euthanasia, tissues were collected and examined for lung, liver, and LN metastases, and each animal was assessed for the number of lung lesions. Differences between the numbers of animals with and without metastases were statistically evaluated by z-test with the level of significance set at p≤0.05. Lymphatic pumping rates resulting from ICG infusion were normalized to that obtained from contralateral sites following i.d. injection and are presented as an average ± SE.

## Abbreviations

Antigens: Ags
CTLA-4: cytotoxic T-lymphocyte-associated antigen-4
HEVs: high endothelial venules
ICG: indocyanine green
i.d.: intradermal
i.m.: intramuscular
irAEs: immune related adverse events
i.v.: intravenously
NIRF: near-infrared fluorescence
LN: lymph node
PD-1: programmed death-1
p.i.: post-implant
s.c: subcutaneous
TDLNs: tumor draining LNs
VAS: visual analog scale
