# Supplementary material for: Nanotopography-based lymphatic delivery for improved anti-tumor responses to checkpoint blockade immunotherapy
Source: Theranostics. 2019 Oct 22;9(26):8332–43. doi: 10.7150/thno.35280 (PMC6857054; doi:10.7150/thno.35280)
Supplement: Supplementary file 1 — Supplementary figures and video legends. [file thnov09p8332s1.pdf]

Supplemental data

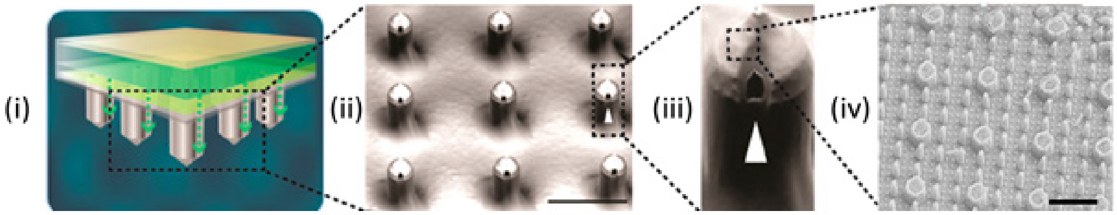

Figure S1. Schematic of the SOFUSA™ Nanotopographical Device (i) The Microfluidic Fluid Block with a perforated attachment adhesive (tan), microfluidic distributor (green), perforated attachment adhesive (yellow), and silicon microneedle array (gray). Each microneedle is 350 µm long, 110 µm wide with a 30 µm through hole located off center which the drug flows out. (ii) Scanning Electron Microscopy (SEM) image of nanotopographic film heat formed over the silicon microneedles (scale bar represents 300 µm) (iii) SEM of individual microneedle, and (iv) SEM image of the nanostructures on each microneedle (scale bar = 3 µm). Adapted from [10].

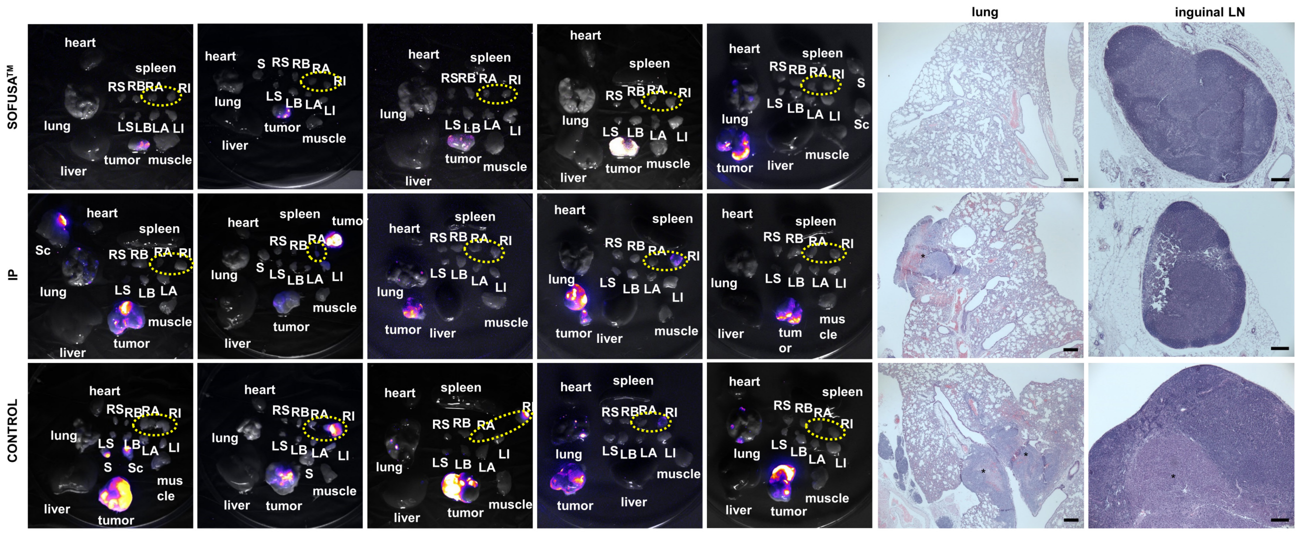

Figure S2. Bioluminescent images of excised tissues in animals treated i.p with anti-CTLA-4 or control antibody, or via SOFUSA[TM] with anti-CTLA-4. Representative H&E images of excised

lung (scale, 200 μm) and inguinal LN (scale, 100 μm). Asterisk, metastatic tumor. RS/LS, right/left submandibular LN. RB/LB, right/left brachial LN. RA/LA, right/left axillary LN. RI/LI, right/left inguinal LN. S, stratum. Sc, scapula. Yellow-dotted oval represents tumor draining inguinal/axillary LNs.

Video 1: Movie of SOFUSA^{TM} delivery of ICG to brachial LN.

Video 2: Movie of active lymphatic propulsion following SOFUSA^{TM} and contralateral i.d. injections in the medial aspect of the calf.
